# Simultaneous Intercalation and Assembly of Graphene Oxide and Polydiallyldimethylammonium Chloride (PDDA) for Hydrogen Purification

**DOI:** 10.1021/acsanm.6c00704

**Published:** 2026-06-16

**Authors:** Giacomo Foli, Vasiliki Benekou, Fabiola Liscio, Roberta Di Carlo, Benedetta Ferrari, Elisa Franzoni, Zhenyuan Xia, Vincenzo Palermo, Matteo Minelli, Marco Giacinti Baschetti

**Affiliations:** † Department of Civil, Chemical, Environmental, and Materials Engineering (DICAM), 9296 University of Bologna, via Umberto Terracini 28, Bologna 40131, Italy; ‡ Institute for Organic Synthesis and Photoactivity (ISOF), National Research Council of Italy (CNR), via Piero Gobetti 101, Bologna 40129, Italy; § Institute of Nanostructured Materials (ISMN), National Research Council of Italy (CNR), via Piero Gobetti 101, Bologna 40129, Italy; ∥ Centro Ceramico, via Valle D’Aosta 1, Sassuolo, MO 41049, Italy; ⊥ Department of Mechanical Engineering, 11248 Chalmers University of Technology, Hörsalsvägen 7B, Gothenburg 412 96, Sweden

**Keywords:** membranes, gas separation, graphene oxide, layer-by-layer assembly, nanostructured architecture

## Abstract

Hydrogen will be the energy vector of our future, but cost-effective production of such gas is still far from being established. Nowadays, major issues in hydrogen synthesis are its cost-effective separation from process byproducts, mainly CO_2_ or CH_4_. Bottom-up fabrication of molecular nanoarchitectures composed of sheets of 2D materials such as graphene oxide (GO) offers a potentially tunable platform to prepare versatile gas membranes able to obtain pure hydrogen for industrial applications. In this work, we assembled a series of PDDA–GO composite nanomaterials with tunable thickness, exploiting the strong interaction between GO and PDDA and varying the number of deposition cycles. Surprisingly, we observed excellent selectivity for hydrogen even after one single deposition cycle, with the membrane ca. 4 nm thick showing the highest permeance. Using extensive characterization with quartz crystal microbalance, atomic force microscopy, X-ray diffractometry, and X-ray photoelectron spectroscopy, we could attribute such an unexpected combination of selectivity/permeance to the formation of an interpenetrated multilayered structure with GO sheets spaced approximately 1.1 nm apart and intercalated by polymer chains, which self-assemble forming an intercalated layered structure even after a single deposition cycle. This approach could significantly reduce the complexity and cost of production for gas separation membranes.

## Graphical abstract

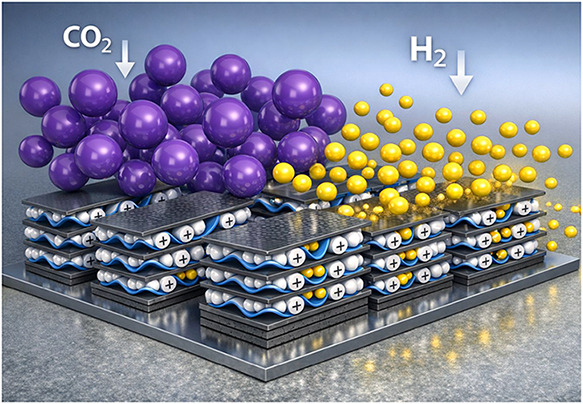

## 1 Introduction

Hydrogen is widely considered the energy vector of the near future.^1^ Compared with conventional fossil-based fuels, hydrogen combustion does not lead to the emission of dangerous chemical species, and only water vapor is produced. At the same time, saving fossil feedstocks from combustion would allow a better use of such resources into production of added value chemicals.^2^ Unfortunately, the absence of molecular hydrogen on our planet forces us to produce it. At present, two main technological routes for hydrogen utilization are viable: gasification and the power-to-gas approach. Gasification uses specific feedstocks, both fossil or renewable,^3,4^ to produce pure hydrogen (H_2_) after its separation from by-produced carbon dioxide (CO_2_). Nevertheless, transportation of hydrogen is not easy, since existing infrastructures have been constructed to deal with natural gas, and a capillary distribution of hydrogen is still challenging.^5^ Thus, a nowadays solution is represented by power-to-gas plants,^6^ as they utilize sustainably produced green hydrogen^7^ and convert it into easier transportable synthetic methane. In this way, the energy vector to be transported is still methane, but produced with a green and renewable process. Once the consumption point is reached, synthetic methane can be in situ gasified into H_2_ and CO_2_.^8^ H_2_ will be used as a carbon-free energy vector, and CO_2_ will be recirculated to power gas plants. CO_2_ will then react with green H_2_ to produce synthetic methane, finally closing the loop. Thus, such a closed cycle requires the development of efficient and sustainable technologies to separate H_2_/CH_4_ and H_2_/CO_2_ gas mixtures.

In the past few decades, cryogenic distillation was the main strategy to achieve separation of gas mixtures;^9^ however, its high energy demand has progressively shifted attention toward alternative approaches, and sorption-based processes targeting specific gas species are nowadays widely preferred.^10^ At the same time, adsorption-based processes, such as pressure swing adsorption (PSA), are also currently investigated for gas separation.^11^

Besides these techniques, membranes for gas purification are arising as extremely promising solutions.^12^ Membranes are indeed less energy-intensive with respect to other technologies, as they do not require thermal regeneration steps as in absorption. For this reason, membranes are also generally simpler and more economical to operate with respect to the PSA process, which is intrinsically discontinuous. In addition to that, membrane modularity usually allows a simple and straightforward scale-up with respect to other separation technologies.^13^

Generally, membranes separate species by exploiting differences in their dimension, acting as sieving systems. In gas separation, the size of gas molecules is typically expressed in terms of their kinetic diameter, related to the probability that such a molecule in a gas will collide with another molecule, causing scattering.^14^ Among all materials, polymers are widely used to prepare membranes because they may act as sieves at the nanoscale. Indeed, the polymer chains form a network with nanometric and subnanometric pores, which can effectively act as a nanodimensioned membrane and separate different gases according to their kinetic diameter. In general, the gas separation performance of membranes is measured through permeance and selectivity.^14^ Detailed definitions of both permeance and selectivity are given below (see the Experimental Section).

Polymeric membranes are widely used for gas separation, as they are cheap and easy to process, but they also have some drawbacks, the main one being related to the trade-off existing between high gas permeance through the membrane and high process selectivity. Such a limit, usually described by the Robeson upper bound,^15^ derives from the random, uncontrolled structure of the polymer macro chain network, causing a wide variability of the dimension of the sieving network.

To overcome such a limitation, the bottom-up fabrication of new nanoarchitectures may be explored, aiming to combine high permeance due to short diffusion paths with high selectivity due to well-defined, nanometric pores. This goal can be achieved by using nanodimensional building blocks to control the structure of the material at the nanoscale and consequently produce membranes suitable for the separation of gases of interest.^16^ Among all suitable building blocks, graphene is probably the most intriguing candidate in the gas separation field.^17^ Its 2-dimensional shape is ideal for the bottom-up assembly of a membrane, it can be produced with a scalable procedure at reasonable costs,^18^ and it is already widely studied in membrane separation application.^16^ In the last few years, gas membranes made of a single layer of graphene^19^ or by more robust multilayers of graphene^20^ have been prepared, but the occurrence of cracks, i.e., loss of selectivity, or extremely low gas permeance have turned out to be major issues toward the successful production of efficient hydrogen separation membranes. Also, nanoporous graphene was successively prepared and used for efficient gas separation by controlling the dimension and concentration of nanopores.^21,22^ All carbonaceous materials allowed good results to be achieved in terms of hydrogen purification using different approaches and morphologies, and sheets of graphene oxide (GO) were used as well.^23^ As a consequence of its preparation, GO sheets feature typically negative surface charges; thus, positively charged species like polycations can be used to foster even further self-assembly of supramolecular nanostructures. As an example, taking advantage of the electrostatic layer-by-layer self-assembly technology,^24^ a vast series of molecular architectures like the ones illustrated above have been successfully prepared in the last few years.^25–27^ Among the different possibilities, polydiallyldimethylammonium chloride (PDDA) turned out to be an ideal polycation for the preparation of such systems.^28,29^ Interaction of positive chains of PDDA with negatively charged GO sheets yields layered PDDA-intercalated graphenic nanoarchitectures, which, when properly designed, can feature a sieving action similar to that of random polymer membranes, but with a more controllable structure and, consequently, better separation performances. Targeting hydrogen purification, the key advantages of such composite membranes not only reside in the high selectivity attainable. For instance, the performance of a layer-by-layer self-assembled membrane could be easily tuned simply by varying the number of deposition cycles. This feature also opens the possibility of integrating such membranes into multistage configurations, where the permeability and selectivity can be tuned to meet the specific requirements of each process stage.^13^

Generally, it is believed that the transport of gaseous molecules in graphenic nanoarchitectures takes place through cavities present on the graphenic layers.^30^ At the same time, the transport along graphenic sheets provides size selectivity, with smaller penetrants that will diffuse faster along the planes. Gas separation with two or even one single layer of graphene has been studied,^31,32^ but this approach typically requires the precise control of nanometric holes in the sheets. Conversely, it is relatively easy to tune the interlayer spacing between stacked nanosheets to allow preferential intercalation of specific molecules or ions.^33–35^ In this approach, it is reasonable to infer that fabrication of a continuous nanoarchitecture, able to impart size selectivity, could be possible only when enough graphenic layers are deposited. On the other hand, overall permeance would benefit from having a lower number of graphenic layers. It is important, therefore, to tune the deposition process to obtain an ordered nanoarchitecture able to guarantee high selectivity with a limited number of graphenic layers, thus leading to a membrane able to surpass the Robeson limit.

For this reason, we investigated here the permeation properties of PDDA-intercalated graphenic nanoarchitectures composed of few graphenic layers, namely, membranes. Using our consolidated approach,^28^ we employed the layer-by-layer deposition method to assemble membranes of GO nanosheets alternated with widely used commercial PDDA. By tuning the number of PDDA–GO deposition cycles, multilayered sieving membranes of different thicknesses were fabricated, as illustrated in Figure 1. We then studied whether these new membranes could have higher permeance than state-of-the-art PDDA–GO membranes described in previous works while maintaining high gas separation selectivity, in particular for H_2_/CO_2_ and H_2_/CH_4_ separation.

**Figure 1:**
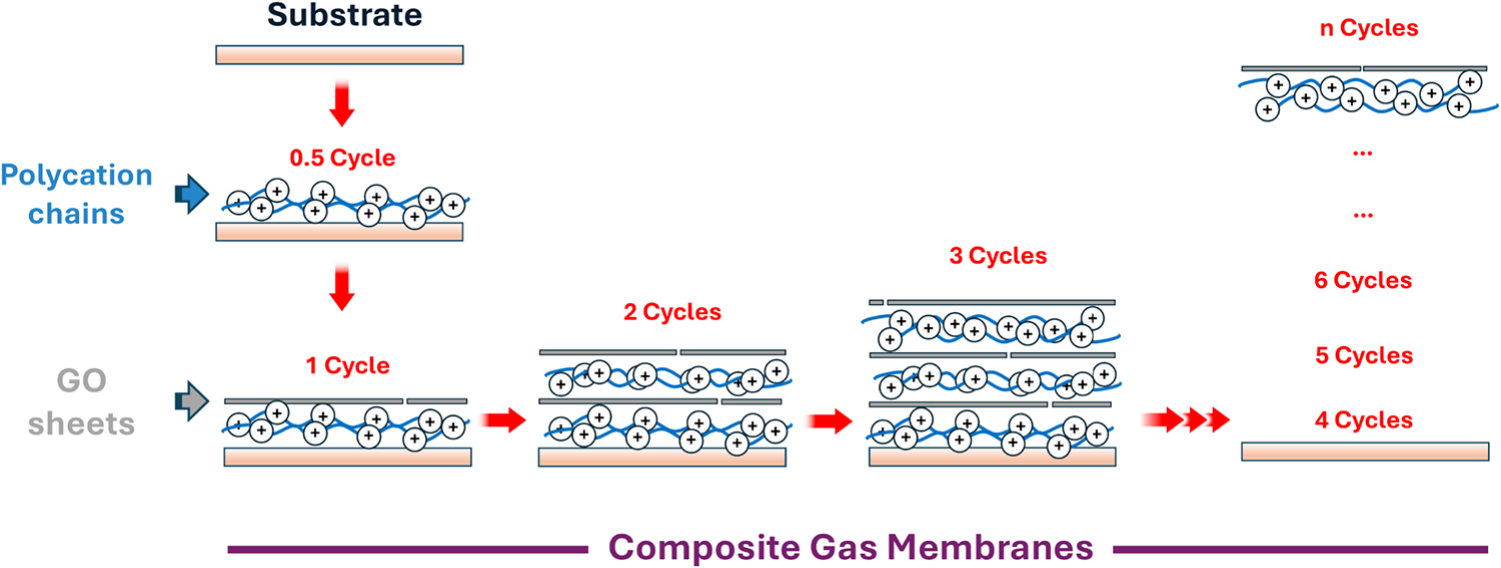
Schematic representation of the fabrication approach of membranes prepared with different numbers of PDDA–GO deposition cycles.

## 2 Results and Discussion

We assembled our membranes by sequential deposition of positively charged PDDA chains and negatively charged GO nanosheets (see the Experimental Section). Our membranes were fabricated from highly hydrophilic materials and were expected to undergo non-negligible swelling under humid conditions, with a corresponding modification of their gas permeation behavior. Although the membranes were stable in water and were extensively rinsed during fabrication, a previous work showed that residual water strongly bound within the alternating layers could significantly influence gas permeability.^36^ In particular, GO–polymer membranes exhibited higher permeability in the as-prepared state, while permeability decreased after removal of residual water via prolonged vacuum treatment and heating (oven and IR lamp). For this reason, all membranes investigated in this work were IR-heated and then dried in a vacuum oven at 35 °C for 10 days prior to permeation measurements. In prospective industrial applications, humidity control will be essential to ensure stable membrane performance. Notably, pretreatment of process streams to remove moisture is already a common operation in industrial practice.^37^ We then evaluated the performance of the PDDA–GO membranes prepared after 10, 5, 3, or 1 cycle of deposition for selective separation of H_2_/CO_2_ and H_2_/CH_4_ gas mixture. Typical outputs of the permeation tests performed with the three penetrants through all of the fabricated membranes are given in Figure S1. The detailed description of the experimental procedure to perform the test on our membranes and the calculation of gas permeance are reported in the SI. All membranes showed effective gas separation properties, exhibiting molecular-size-dependent transport behavior; as could be expected, larger molecules like CH_4_ and CO_2_ presented lower values of permeance compared to H_2_, and thinner samples, i.e., fewer deposition cycles, showed higher permeance, spanning from ca. 0.21 to more than 20 GPU for H_2_ (Figure 2a). We also verify the stability of permeation values under the influence of temperature changes by permeating H_2_ through the 10 cycles of deposition membrane at increasing temperature (Figure S2). Permeance values regularly increase with temperature, suggesting that the permeation process through the membrane is predominantly diffusion-controlled.^14^ Most importantly, the final lowering of the temperature of permeation to the starting value, i.e., 35 °C, resulted in the calculation of a permeance well comparable with the original value: 0.24 ± 0.02 GPU vs 0.21 ± 0.05 GPU (original value).

**Figure 2:**
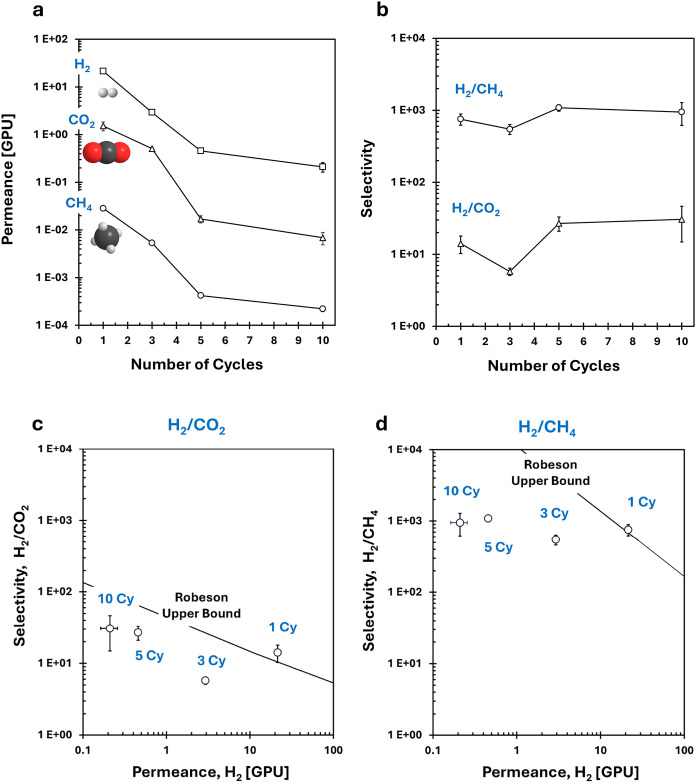
(a) Gas permeance values in the PDDA–GO membranes as a function of the number o deposition cycles, and (b) resulting selectivity for the two gas mixtures considered; the performances of the membranes prepared with 1, 3, 5, and 10 cycles of deposition (Cy) are reported in the Robeson plot of (c) H_2_/CO_2_ and (d) H_2_/CH_4_ (in (c, d), the Robeson limit is reported as a solid black line).

The separation performance, measured as selectivity, of these membranes was very good in all samples, with H_2_ diffusing ca. 1000 times faster than CH_4_ and ca. 10 times faster than CO_2_ (Figure 2b). Notably, while permeance increased moving from thicker to thinner membranes, reaching a value of 21.4 ± 1.4 GPU for H_2_ for 1 cycle of deposition, selectivity remained substantially constant. Such high selectivity combined with high H_2_ permeance allowed our thinner membrane to outperform thicker ones, exceeding the current state of the art (Robeson limit) in separation of both H_2_/CO_2_ and H_2_/CH_4_ gas mixtures (Figure 2c,d). All of the permeation results presented herein are the average of at least three independent tests performed for the three penetrants toward each of the four membranes (see Table S1).

In previous works,^28,29^ we discussed that selectivity was mainly due to differential diffusion of gases in the space between different GO sheets, filled by polymer chains, but a system made of a single PDDA layer with a single GO layer on top would not be able to show such selectivity, and some other mechanism should be in place. Hence, we decided to fully characterize our membranes and point out the origin of this unexpected behavior.

The composite nature of our membranes was investigated by X-ray photoelectron spectroscopy (XPS), which was used to confirm the presence of PDDA in the membrane and its interaction with GO. In more detail, as shown in Figure 3, the presence of the nitrogen signal in the XPS spectrum of the membrane (N signal ca. 400 eV) unquestionably proved the presence of PDDA. More importantly, the absence of the original counterion of PDDA, i.e., chlorine, in the spectrum of the membrane (Cl signal ca. 200 eV) guaranteed the achievement of a new macromolecular architecture. Indeed, chlorine exchanged with the functional groups of GO during self-assembly, resulting in the fabrication of our membrane.

**Figure 3:**
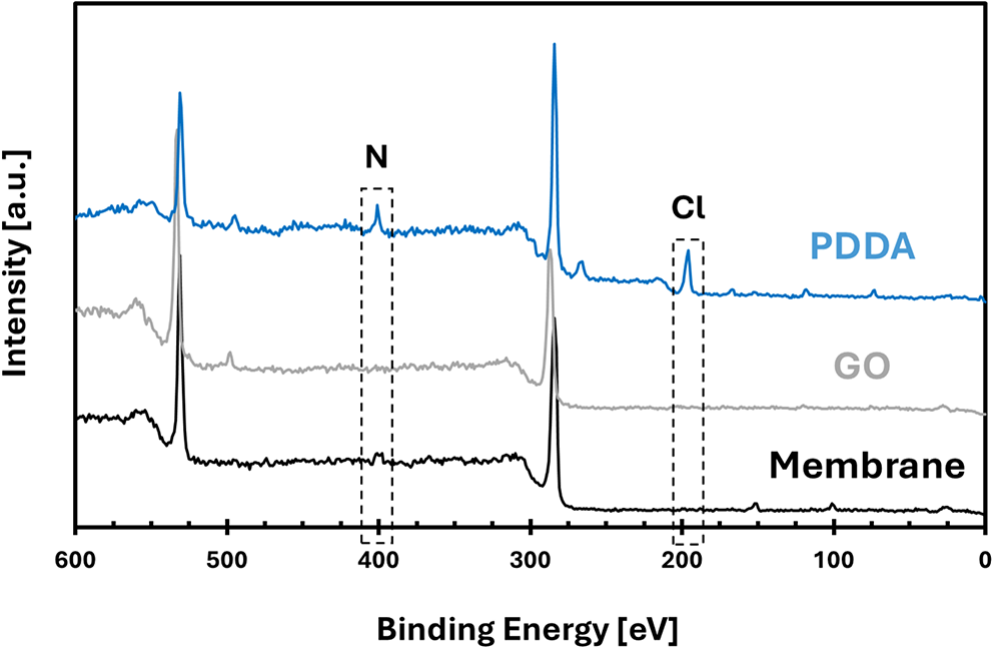
XPS spectra of PDDA, GO, and the 10 cycles of deposition membrane.

Scanning electron microscopy (SEM) of the top surfaces of the four membranes confirmed the presence of GO sheets (see Figure 4). For the sake of completeness, we also recorded the SEM of the flat polyimide substrate on which the membranes were self-assembled (see Figure S3 and the Experimental Section for details).

**Figure 4:**
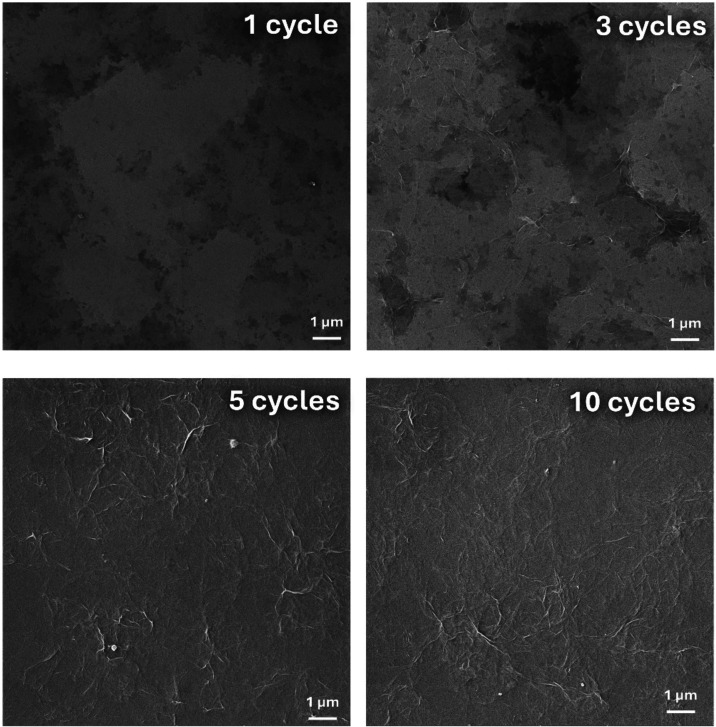
SEM of the top surface of the four fabricated membranes.

The deposited sheets of GO were clearly visible after 10 and 5 cycles of deposition, as one can clearly see from the wrinkles present on the top surface of these two samples. Nevertheless, graphenic sheets were also observable after 3 and even after only 1 cycle of deposition, but morphologies were appreciably different. To quantify such differences, we decided to study the superficial roughness of the surfaces of our membranes by optical profilometry (OP) and atomic force microscopy (AFM). Figure 5 shows OP and AFM characterization of the surface of the membrane obtained after 10 cycles of PDDA–GO deposition. Notably, the RMS roughness measured by OP and AFM gave comparable values of 5.4 ± 0.6 and 5.5 ± 0.5 nm, respectively. Similar morphologies were also obtained on thinner membranes prepared after 5, 3, and 1 cycles of deposition, even with lower roughness. Table 1 compares RMS roughness for different samples, with an increase in the number of deposition cycles yielding an increase in roughness: all acquired images (AFM and OP) are reported in the SI (Figures S4 and S5, respectively).

**Figure 5:**
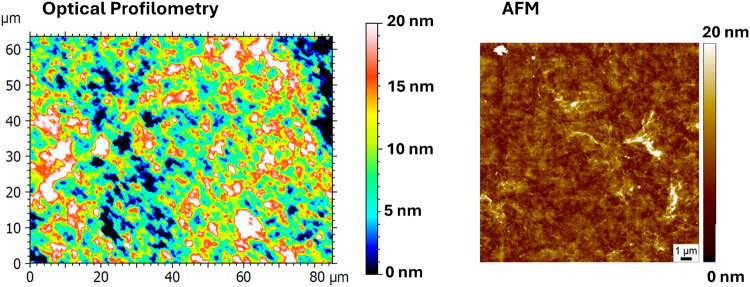
Surface morphology of the samples prepared with 10 cycles of deposition acquired with an optical profilometer (OP), on the left, and AFM, on the right.

**Table 1:** Root Mean Square Roughness (RMS) Calculated for the Substrate and the Membranes Prepared after Various Cycles of Deposition from Optical Profilometry and AFM Data^a^

	RMS, [nm]	
	**optical profilometry**	**AFM**
substrate	1.2 ± 0.1	0.8 ± 0.3
1 cycle	2.1 ± 0.1	1.4 ± 0.1
3 cycles	2.5 ± 0.1	2.7 ± 0.2
5 cycles	3.6 ± 0.8	4.3 ± 0.3
10 cycles	5.4 ± 0.6	5.5 ± 0.5

a For the substrate and the 1 cycle of deposition membrane, differences in roughness are due to the different areas sampled by the two techniques: 85 × 63 μm^2^ for OP and 20 × 20 μm^2^ for AFM.

Morphological analyses highlighted that, even after a few cycles of deposition, a continuous structure was fabricated. For this reason, we decided to study the PDDA and GO deposition process using a quartz crystal microbalance (QCM). Such a technique measured mass changes and surface interactions on a quartz crystal sensor by monitoring its resonance frequency shift during deposition cycles. The equation used to convert the frequency shift in mass change is reported in the SI. In this work, we calculate the mass changes during the fabrication of three independent membranes with 10 deposition cycles. To generalize the data, we rescaled mass changes over the active area of the used sensor, achieving stepwise increases in grammage, i.e., the amount of the material deposited and measured in μg · cm^–2^. All of the calculated stepwise increases are reported in Table S2. For experimental details, see the Experimental Section.

Figure 6a shows that the self-assembly of the PDDA–GO membranes follows a linear growth regime,^38^ with a regular step-by-step increase in grammage observed after each alternated deposition of PDDA and GO (circle and diamond, respectively, Figure 6b). In both cases, a constant amount of the material was deposited at each step: 0.14 ± 0.05 μg·cm^–2^ for PDDA and 0.46 ± 0.12 μg·cm^–2^ for GO. Such a constant amount of the material was already deposited in the very first cycle, making the observed permselectivity for the 1-cycle membrane more plausible, as reported above. Notably, the amount of GO deposited at each cycle was much more than what would be expected for the deposition of a monolayer. Indeed, using an ideal GO density of ≈1.8 g·cm^–3^,^39^ the reported grammage corresponds to a GO equivalent thickness of ca. 2.56 nm, while a single GO layer is ≈0.8 nm thick. This suggests that a thicker multilayer of GO sheets was deposited at each cycle. Thus, we used AFM to measure the thickness of the material deposited on a flat substrate (see the Experimental Section and Supporting Information) after 1, 3, 5, and 10 PDDA–GO cycles of deposition (Figure 7). Details of the calculation are available in the SI (also see Figure S6 and Table S3).

**Figure 6:**
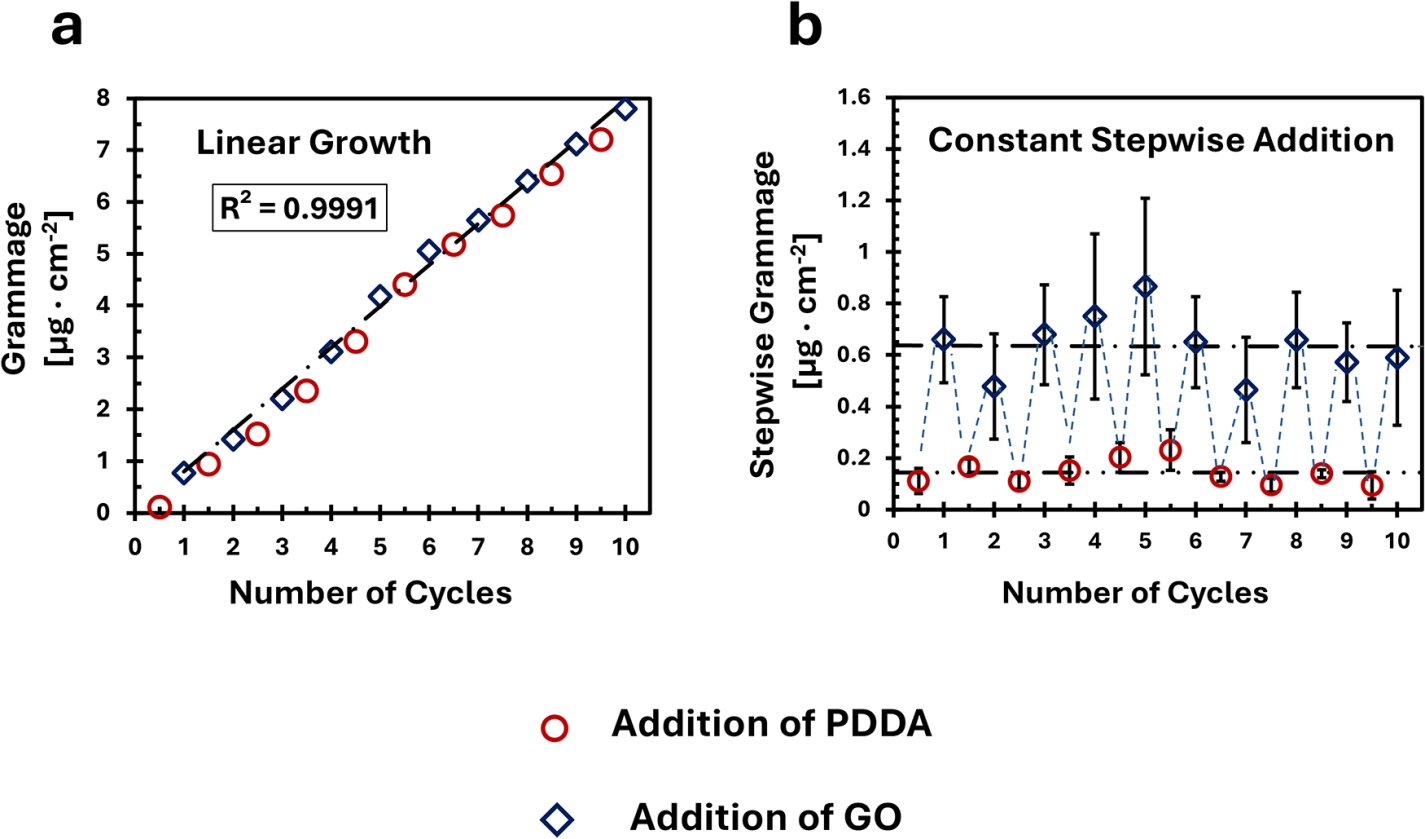
(a) Linear growth regime of the membrane for each half-cycle (PDDA on top, circle) and complete cycle (GO on top, diamond). (b) Constant stepwise increase in grammage recorded for the alternated exposure of the sample to PDDA (circle) and GO (diamond) solutions.

**Figure 7:**
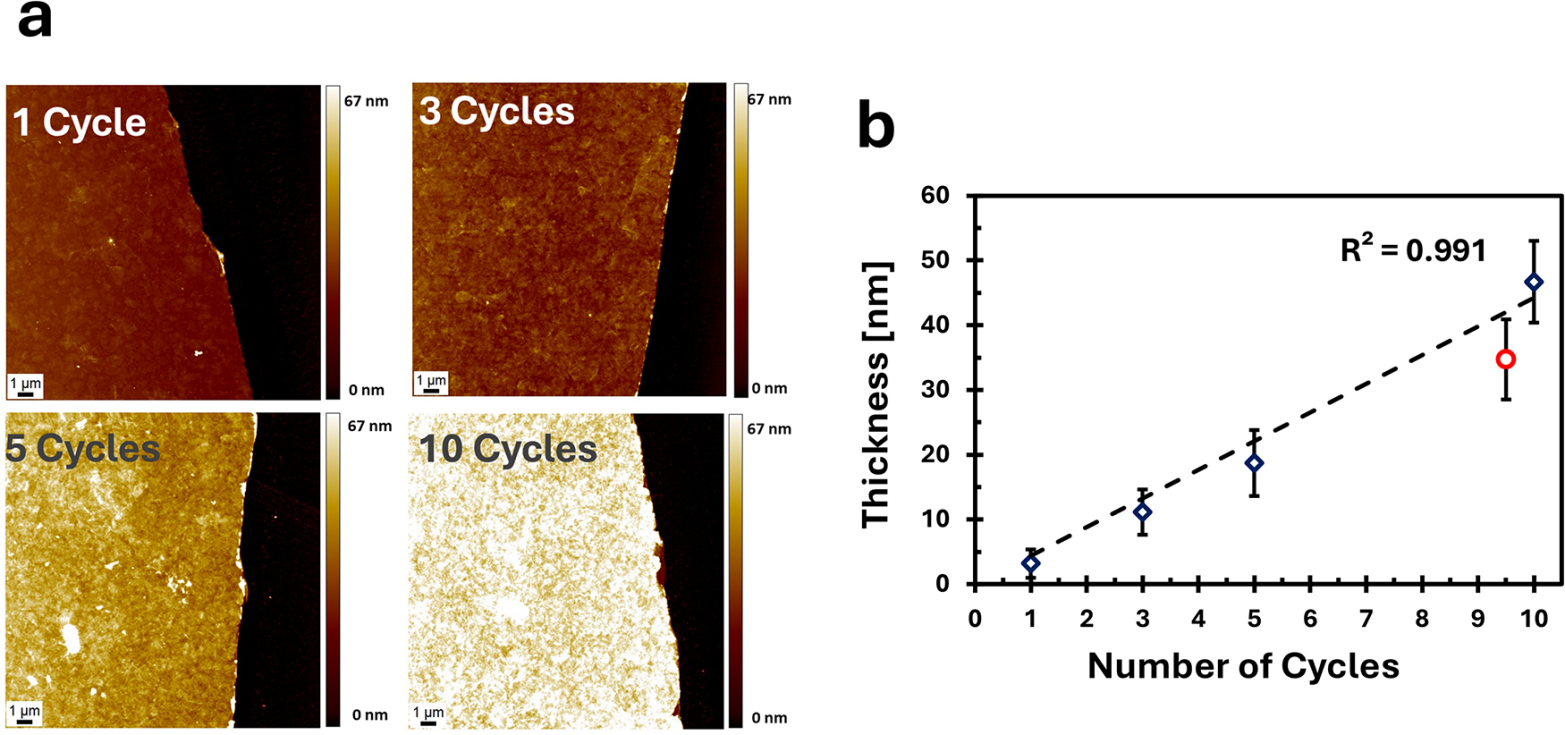
(a) AFM images of the membrane after 1, 3, 5, and 10 cycles of deposition growth on the flat substrate. The coating has been scratched on the right part of the image to measure its thickness. The *z*-range of all images has been fixed to 67 nm to better compare the increase in thickness. (b) Thickness of the membranes upon an increase in the number of PDDA–GO cycles of deposition (diamonds). The thickness measured for a sample missing the last GO deposition is also shown as a red circle. The substrate thickness has been removed for clarity (see Figure S7a and Table S3).

Using the results obtained after 10 deposition cycles, an average thickness L of 4.7 ± 0.6 nm was calculated for each cycle of deposition, and this value was quite constant. Notably, the thickness of the architecture prepared after 9.5 cycles, i.e., a self-assembly of 9 cycles of PDDA–GO, followed by a final half-cycle made of only PDDA, was in accordance with other results (Figure 7b, circle, and Figure S7b). Indeed, the cycle-rescaled average thicknesses were comparable: 3.7 ± 0.7 nm (9.5 Cy) vs 4.7 ± 0.6 nm (10 Cy) (see Table S3). Combining this value with the total mass of PDDA–GO deposited per cycle, 0.60 ± 0.17 μg·cm^–2^ measured by QCM, we could estimate the density of our membrane as 1.26 g·cm^–3^. This value was significantly lower than that of bulk GO (≈1.8 g·cm^–3^),^39^ and the difference could be attributed to the presence of PDDA, characterized by a bulk density of 1.21 g·cm^–3^ (see the Experimental Section). Thus, AFM and QCM combined data suggested that more than one GO layer was deposited at each cycle. Relevantly, the deposition of negatively charged GO should be self-limiting due to the repulsion between negatively charged GO sheets; thus, some counterions should be involved in the process, intercalating in between GO and allowing deposition of a multilayer in a single cycle of deposition. This evidence also agreed with the estimated density of the deposited layer, lower than bulk GO, and suggested that the final material is not a simple sequence of segregated, well-defined PDDA and GO phases deposited on top of each other at every cycle of deposition, but rather an electrostatic-driven assembly of the two components.

We used XRD measurements to inspect the presence of periodic intercalated structures in the samples. In Figure 8, a peak at 2θ = 8.1 ± 0.1°, which corresponds to a *d*-spacing of 1.09 ± 0.06 nm, could be observed for all of the specimens investigated, with the exception of the first cycle of deposition. The intensity of this peak increased with the number of deposition cycles used to self-assemble the membrane. The spacing is slightly lower with respect to the value observed in our previous work, but this could be ascribed to the more negative ζ-potential of GO used in this work: −58.3 ± 8.0 vs −37.8 ± 5.7 mV (see Table S4). Even the atomic O/C ratio determined by XPS agreed with such a conclusion: 0.51 (this work, see Figure S8) vs 0.38 (previous work).^28^ Finally, the observed *d*-spacing in our membranes was significantly larger than that of pristine GO (2θ = 11.05 ± 0.02°, *d* = 0.89 ± 0.08) (see Figure S9 in the SI).

**Figure 8:**
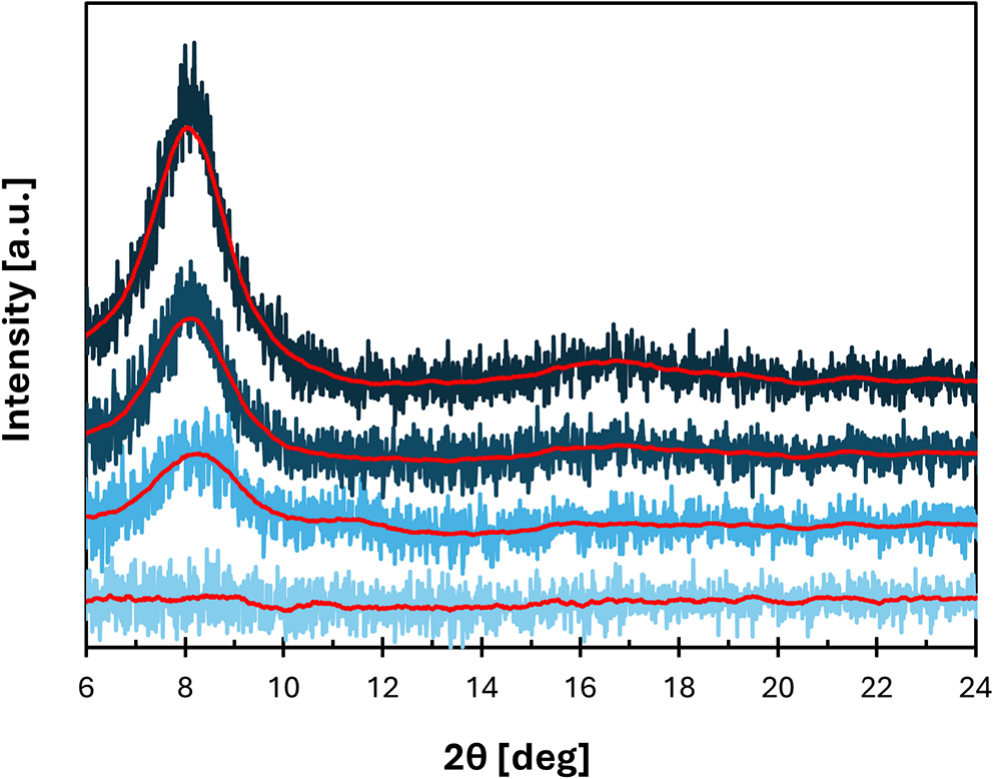
XRD patterns collected for the membranes, from bottom to top: after 1, 3, 5, and 10 cycles of deposition; smoothed red lines are shown as visual aids.

It is well known that GO intersheet spacing can increase due to absorption of water.^40^ Thus, we performed some comparative tests to exclude that the larger *d*-spacing of the membrane was not related to the intercalation of residual water molecules from the layer-by-layer deposition. Heating in an oven at 35 °C for several days and further treatment at 100 °C in vacuum for 1 h did not affect the spacing of our PDDA–GO membrane (see Table 2, entries 1–2, and Figure S10). On the contrary, hydration of neat GO effectively increased the value of *d*-spacing, but the only heating in an atmospheric oven at 35 °C restored the original value in a few days (Table 2, entries 3, 4, and 5, and Figure S9).

**Table 2:** XRD Main Peaks for the Different Samples Analyzed

entry	sample	*d-*spacing [nm]
1	PDDA–GO membrane	1.09 ± 0.06
2	PDDA–GO membrane, dried	1.10 ± 0.08
3	pristine GO	0.80 ± 0.08
4	GO swollen in water (wet GO)	1.06 ± 0.04
5	GO swollen in water, dried	0.79 ± 0.04
6	GO swollen in PDDA water solution, dried in an oven	0.91 ± 0.01 (partially intercalated) 0.82 ± 0.02 (not intercalated)

To verify the possibility that PDDA chains could effectively interact with GO sheets and generate a multilayered PDDA-intercalated architecture, we studied the interaction of GO sheets and PDDA in solution. We prepared a concentrated suspension/solution of GO (9 mg/mL) and PDDA (10 mg/mL) in water by sonication. After sonication, the mixture was stored in the dark for 10 days, and the GO powder was separated from the supernatant PDDA solution by centrifugation. The resulting powder was then dried in vacuum at 35 °C for an additional 10 days. The XRD of the dried powder showed a main peak corresponding to *d* = 0.91 ± 0.01 nm and a shoulder at *d* = 0.82 ± 0.02 nm, corresponding to partially intercalated and pristine GO, respectively (see Table 2, entry 6, and Figure S9). Thus, intercalation of PDDA into GO layers can also effectively take place in solution, even if only partially, yielding two different peaks likely due to slow penetration of PDDA in the GO flake powder. Conversely, our cycled deposition could yield much better interactions of GO and PDDA, with XRD showing only a single peak at large spacing (*d =* 1.09 ± 0.06 nm).

We used Scherrer’s formula^41^ to estimate the average thickness of the ordered domains in our membrane from the XRD patterns: *L*′ = 4.7 ± 0.2 nm (see Table S5). This value agreed well with the above-reported AFM result, *L* = 4.7 ± 0.6 nm, which represented the increase in the thickness of the membrane after each deposition cycle. Consequently, the relatively sharp XRD peaks generated by our membranes at *d* ≈ 1.1 nm corresponded, via Scherrer analysis, to coherent domains with a thickness *L*′ of ≈4–5 nm, in very good agreement with the thickness increment per deposition cycle *L*, measured by AFM. This showed that each PDDA–GO cycle of deposition did not produce two separate layers, one PDDA and one GO phase, but a single intercalated block of thickness ≈4–5 nm, in which PDDA chains and GO sheets mix and stack together. Each block was therefore a multilayered PDDA–GO domain, consisting of several GO sheets separated by PDDA, with an average thickness of about 4–5 nm and an interlayer spacing of 1.1 nm. AFM also indicated that a similar thickness increment had already occurred in the sample prepared with only 1 cycle of PDDA–GO deposition (Figure 7). In this case, however, the total amount of material was too low to generate a detectable XRD peak (Figure 8).

With an increasing number of deposition cycles, the coherent domain thickness extracted from Scherrer analysis slightly increased, while the broadening of the corresponding rocking curves (see Figure S11) indicated a growing distribution of orientations of these PDDA–GO blocks with respect to the substrate normal. This trend was consistent with the progressive increase in surface roughness observed by OP and AFM (Figure 5 and Table 1), suggesting that additional deposition cycles led to more corrugated and partially misoriented stacked domains, increasing the mosaicity of the architecture.^42^ These last observations allowed us to postulate that every single cycle of PDDA–GO self-assembly creates a complex, multilayered structure: a schematic representation of the mechanism of a single cycle of deposition is reported in Figure 9.

**Figure 9:**
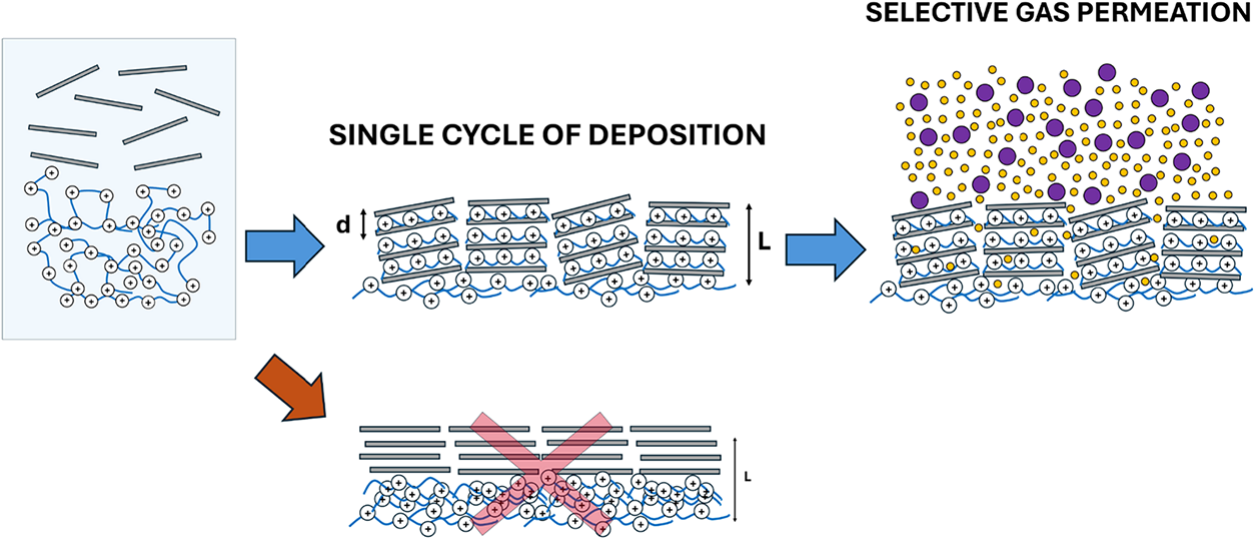
Schematic representation of the proposed self-assembly mechanism of a single cycle of deposition to prepare the membrane for selective gas permeation.

Microscopy and spectroscopy data consistently demonstrated that the formation of PDDA–GO membranes for gas separation did not follow a simple sequential layer-by-layer deposition of PDDA polymer chains and GO sheets. Due to the strong electrostatic interaction between negatively charged GO and positively charged PDDA, polycation chains intercalated GO sheets during each cycle of deposition. Such a self-assembly process formed highly ordered structures with a measured thickness of approximately 4 nm per cycle, confirmed by AFM and XRD, where GO sheets were uniformly spaced by ca. 1.1 nm, as determined by XRD. The GO sheets strongly interacted with PDDA polymer chains, leading to the complete displacement of PDDA’s chloride counterion (Figure 3).

This distinct intercalated structure is clearly visible via XRD analysis in thicker samples prepared with 3, 5, or 10 cycles of deposition. Unfortunatley, clear XRD data could not be obtained for thinner sample, single-cycle deposition, but this membrane exhibits similar, or even superior, gas separation performances compared to thicker ones (see Figure 2). This suggests that even a single cycle of PDDA–GO deposition could produce a layered structure, where PDDA and GO are mutually intercalated, providing the observed gas selectivities. Thus, the superior performances observed in thinner membranes could be explained by assuming that a single cycle of deposition triggers the self-assembly of a crystalline multilayer consisting of 3 to 4 PDDA–GO-intercalated layers. Such a conclusion was also supported by the measurement of the thickness and mass of the 1-cycle membrane, determined by AFM and QCM, respectively. The above-mentioned increase in mosaicity and surface roughness with the number of cycles of deposition suggests a progressive misorientation of PDDA–GO domains and the formation of interdomain voids. Such defects are expected to act as nonselective shortcuts for gas transport and therefore to decrease, rather than enhance, the intrinsic size-sieving selectivity of the PDDA–GO galleries. The high selectivities observed even for single-cycle membranes thus indicate that gas permeation is dominated by transport within the well-defined interlayer galleries of the intercalated PDDA–GO multilayer, rather than by large defects. The absence of an XRD pattern for the 1-cycle membrane did not allow us to record its rocking curves.

This behavior is consistent with molecular sieving in narrow PDDA–GO slit pores, whose effective width is tuned by the amount and arrangement of intercalated PDDA, which in turn depends on the defect density and oxidation level of the specific GO used.

## 3 Conclusion

We proposed the utilization of an additive self-assembled PDDA-intercalated graphenic nanoarchitecture as a membrane for the purification of H_2_ from its mixture with CO_2_ or CH_4_. By permeating different penetrants, we proved that such an assembly procedure allowed us to prepare graphenic-layered nanoarchitectures to separate different gas molecules, depending on their size. Such architectures, namely, membranes, consisted of a continuous polymeric phase in which sheets of GO were orderly present with an interlayer of 1.1 nm. The diffusing gas molecules had to access such a confined polymeric phase and undergo a nanometric tortuous path to permeate our membranes. The bigger the molecule was, the more the tortuous path slowed down the permeation rate of the molecule itself, resulting in the observed superior values of selectivity. Specific interaction between the polymeric phase, i.e., PDDA, and the tested penetrants could be excluded considering previously published results. Indeed, a PDDA multilayer self-assembled using a polyanion in place of GO had no consequences on the values of selectivity.^28^ By lowering the number of cycles of deposition and preparing thinner membranes, we could observe that, different from most conventional gas separation materials, the increase of permeance did not imply a decrease of selectivity. Consequently, even permselectivities of the membrane fabricated after only one cycle of PDDA and GO deposition could surpass the performance of state-of-the-art materials.^15^ We thus used AFM, XRD, and XPS to study the structure of the material, observing that already during a single PDDA–GO cycle of deposition, a layered structure was formed, featuring ordered domains with a size up to ca. 4 nm. This architecture enables efficient separation of H_2_/CO_2_ or H_2_/CH_4_, thereby exceeding the actual performance in terms of permselectivity.

## 4 Experimental Section

### 4.1 Reactants, Materials, and Sample Preparation

Main reactants were purchased from the following companies and used without further purification: sodium hydroxide and potassium chloride (NaOH and KCl, ACS reagent grade) from Sigma-Aldrich; acetone, iso-propanol, hydrochloric acid 37 wt % (HCl), chloroform (CHCl_3_), dichloromethane (CH_2_Cl_2_), and 1,1,2,2-tetrachloroethane (TCE) from Merck (ACS reagent grade); and *n*-decane was purchased from Tokyo Chemical Industry. Deionized water (DI water, conductivity <0.01 μS · cm^–1^) used in this work was produced by an OSMODEMI 4 Standard (Idrotecnica, Water Purification Systems, Italy), equipped with a reverse osmosis module, and a cartridge packed with mixed-bed ion-exchange resin was used. A 20 wt % water solution of PDDA was purchased from Aldrich, while GO powder was purchased from LayerOne. After proper dilution (final concentrations: PDDA 1 wt %, GO 0.01 wt %), the PDDA solution was used without further operations, while the GO suspension was sonicated for 4 h at 30–35 °C using a 4 L sonication bath (Bandeline Sonorex RK 103 H). Matrimid 5218 (polyimide) was kindly provided by Huntsman Corporation and thermally treated in vacuum at 200 °C for 18 h before usage. Boron-doped silicon (Si) wafer discs, purchased from Silicon Materials Inc., were cut in rectangular pieces (1 × 2 cm) and washed in acetone (sonication bath, 30 min at 60 °C) and then in iso-propanol (sonication bath, 30 min at 60 °C). At the end of the wash, wafers were dried with a nitrogen flux and rapidly spin-coated with polyimide for the fabrication of flat polyimide substrates using a Model WS-650Mz-23NPPB spin coater by Laurell Technologies Corporation. See the section below for the spin coating procedure.

#### 4.1.1 Preparation of the Substrates for the Gas Permeation Analysis (Polyimide Film)

All membranes were self-assembled on polyimide films prepared by solubilizing thermally treated polyimide (see above) in CH_2_Cl_2_ (1.5 wt %). 20 mL of polyimide solution were placed in a glass Petri dish (diameter = 9 cm), and the solvent slowly evaporated. The as-prepared yellow disks (thickness ca. 40 μm) were used as substrates for PDDA–GO self-assembly.

#### 4.1.2 Self-Assembly Fabrication of the Membranes (Layer-by-Layer)

We used an automated dip-coating apparatus following a procedure previously reported.^28^ All membranes were prepared by dipping the substrate (polyimide film) in a 1 wt % PDDA water solution for 5 min. The excess of deposited PDDA was removed by a 20 min rinse in DI water. Then, the substrate was dipped in a GO water suspension (0.01 wt %) for 5 min and, once again, excess of deposited species was removed with a 20 min rinse in DI water. By repeating this procedure *n* times, the *n* cycles of deposition membrane was prepared. After the last cycle of deposition, all of the membranes were heated for 30 min with an IR lamp, keeping the sample temperature below 54 °C, and then stored in vacuum at 35 °C for 10 days before carrying outgas transport analyses. This was made to achieve reproducible results in terms of gas transport rates.

#### 4.1.3 Fabrication of the Flat Polyimide Substrate for Structural Characterization of the GO–PDDA Membrane

The thermally treated polyimide (see above) was solubilized in TCE (0.5 wt %) by overnight stirring at room temperature, until a homogeneous mixture was achieved. Then, rectangular Si wafers were spin-coated with a thin film of polyimide. Specifically, a freshly cleaned Si wafer (see above) was placed in the spin coater, completely covered with polyimide solution in TCE, and spun at 4000 rpm for 30 s. The coated wafers were heated in vacuum at 40 °C overnight. The membranes were deposited on the flat polyimide substrate using the previously described procedure.

#### 4.1.4 Preparation of PDDA-Intercalated GO from Solution (GO/PDDA Mix)

A bulk amount of GO (430 mg) was added to 50 mL of a concentrated water solution of PDDA (10 wt %) and vigorously stirred with a vortex mixer. After interruption of the stirring, the suspension was stored in the dark, and the suspended GO powder was allowed to sediment for 7 days. Then, the supernatant solution was collected, and the PDDA-intercalated GO powder was washed with 25 mL of pure water by centrifugation, then dried in a vacuum oven at 35 °C for 10 days, and measured by XRD. A parallel control experiment was performed in the same way but using pure water instead of the PDDA_(aq)_ solution. The spectra are shown in Figure S9.

### 4.2 Characterizations

#### 4.2.1 Gas Transport Analysis

All gas permeation tests were performed at 35 °C according to the manometric technique reported in ASTM D 1434,^43^ using pure gases. All samples tested had an area of 2.2 cm^2^, and the applied pressure difference (Δ*p*) was in the range of 1.7–1.9 bar; higher Δp values, in the order of 4 bar, were only used in the case of methane in order to increase the flux. Such a difference is not considered to significantly affect performance results, as methane solubility is too low in this condition to cause any change to the membrane structure. All of the permeance (*Q*) values were expressed in the Gas Permeation Unit (1 GPU = 3.35 × 10^–10^ mol m^–2^ s^–1^ Pa^–1^) and measured the gas flux per unit area obtained with unit transmembrane pressure (see eq 1).

1 $$Q=\frac{J}{\Delta p}$$

where *J* was the experimentally determined steady-state flux density, and Δ*p* was the difference in pressure, i.e., the driving force of the gas transport process. Selectivities (α_*i,j*_.) defined the efficiency of the membrane separation process through the ratio of the permeance values of two specific species *i* and *j*.

2 $$\alpha_{i.j}=\frac{Q_{i}}{Q_{j}}$$

#### 4.2.2 XPS

The measurements were carried out at Chalmers University of Technology using a PHI 5500 spectrometer (PerkinElmer, Waltham, MA, USA). The instrument was operated with a monochromatic Al Kα radiation source (1486.6 eV), and the analysis was performed using a 100 μm beam size. The spectrum was calibrated with the C 1s peak at 284.5 eV.

#### 4.2.3 Scanning Electron Microscopy

The morphology of the surface of the multilayers was analyzed by SEM, using a field emission gun instrument (Tescan Mira3, Brno, Czech Republic) equipped with energy dispersive spectrometry (EDS, Bruker probe). All of the SEM samples were made conductive by evaporation of gold before observation (Quorum Q150R ES + coater, Laughton, UK).

#### 4.2.4 Optical Profilometry

An optical profilometer (Leica Dual Core Microscope 3D DCM, Leica Microsystems S.r.l., Milano, Italy) was used to evaluate the average surface roughness (Sa) of the membranes. For each sample, at least four surface areas measuring 85 × 63 μm were acquired, using a 150× confocal objective. Sa values are extracted from the acquired areas according to ISO 21920–2,^44^ using the dedicated software (MountainsMap software, version 10, Digital Surf, Besançon, France).

#### 4.2.5 Atomic Force Microscopy

The AFM imaging was obtained by a Multimode 8 microscope equipped with a Nanoscope V controller and a type J piezoelectric scanner (Bruker, USA). Topographic images were obtained in PeakForce mode using ScanAsyst-Air probes (Bruker, USA) in air, imposing an applied force of 2.5 nN. Antimony-doped silicon probes (Bruker RTESPA, cantilever length of 125 μm) with a nominal tip radius of curvature of 8 nm, a nominal spring constant of 40 N/m, and a resonance frequency range of ∼300 kHz were used for all measurements. Images were captured at a scanning rate of 1.0 Hz and resolution ranging from 512 pixels × 512 pixels to 1024 pixels × 1024 pixels. We performed image processing to correct piezo-scanner artifacts with SPIP software. The SPIP software values were used throughout this study for processing AFM images, obtaining the Sa roughness (average roughness). The average roughness Sa was calculated and averaged from 2 acquired images of identical scan size for each sample. The AFM images shown in the main text were leveled by a first-order line prior to any analysis; no further filters were applied.

#### 4.2.6 Quartz Crystal Microbalance (QCM) Analysis

QCM data were collected using a WinQCM fabricated from ElbaTech (Marciana, Italy) equipped with a poly­(methyl methacrylate) fluidic cell: electric signals were acquired with a software supplied by the producer, WinQCM build 2.18. All analyses were recorded using a 10 MHz quartz crystal metalized with gold (quartz diameter: 13.95 mm, gold active area: 0.348 cm^2^) purchased from Industria Elettronica Varese (Varese, Italy). Prior to each analysis, all crystals were spin-coated with a thin film of polyimide with the same spin coating procedure used for Si wafers (see above). To simulate the deposition procedure, the coated quartz was first exposed to a 1 wt % PDDA_(aq)_ solution for 5 min, then washed with deionized water, and, after 20 min, dried with a flux of anhydrous air (flux: 10 Standard Cubic Feet per Hour of Air). The change in frequency was recorded, and the same procedure was repeated using a 0.01 wt % GO_(aq)_ suspension instead of the PDDA solution.

#### 4.2.7 XRD

The measurements were performed using a Rigaku SmartLab (Tokyo, Japan) diffractometer equipped with a rotating Cu anode (λ = 0.1254 nm) and a HyPix-3000 2D detector used for 1D scanning. Powder diffraction was performed in Bragg–Brentano geometry, whereas specular scans and rocking curves were carried out on membranes in parallel beam geometry.

#### 4.2.8 Determination of the Bulk Density of PDDA

The density of PDDA (dried from commercial water solution) was determined using an analytical scale with an accuracy of 0.01 mg (type: NewClassic MF, model: MS105DU, Mettler Toledo, Switzerland) equipped with a density kit (MS-DNY-54, Mettler Toledo, Switzerland). The liquid used for the hydrostatic measurement was *n-*decane, and the values of density reported above were the average of three independent measurements.

#### 4.2.9 Determination of the Superficial ζ-Potential

All ζ-potentials were recorded at pH = 7 in a 1 mM aqueous solution of KCl, using an electrokinetic analyzer (SurPASS 3, Anton Paar, Austria) equipped with an adjustable gap cell (dimension: 10 × 20 mm).

## Supplementary Material



## Notes

Additional experimental details; experimental data sets and equations used to calculate all of the average values presented in the paper; all of the images acquired for morphological characterizations and measurements of roughness (SEM, AFM, and OP); XPS spectrum on neat GO used in this work; and XRD patterns used for the final considerations on the proposed mechanism of self-assembly (PDF)
