# Supplementary material for: Simultaneous Intercalation and Assembly of Graphene Oxide and Polydiallyldimethylammonium Chloride (PDDA) for Hydrogen Purification
Source: ACS Appl Nano Mater. 2026 Jun 16;9(25):11678–89. doi: 10.1021/acsanm.6c00704 (PMC13317670; doi:10.1021/acsanm.6c00704)
Supplement: Supplementary file 1 [file an6c00704_si_001.pdf]

Supporting Information

Simultaneous intercalation and assembly of graphene oxide and poly diallyl dimethyl ammonium chloride (PDDA) for hydrogen purification

*Giacomo Foli, * [a] Vasiliki Benekou, [b] Fabiola Liscio, [c] Roberta Di Carlo, [a] Benedetta Ferrari, [d] Elisa Franzoni, [a,d] Zhenyuan Xia, [e] Vincenzo Palermo, [b,e] Matteo Minelli, [a] and Marco Giacinti Baschetti [a]*

[a] Department of Civil, Chemical, Environmental, and Materials Engineering (DICAM), University of Bologna, via Umberto Terracini 28, Bologna 40131, Italy

[b] Institute for Organic Synthesis and Photoactivity (ISOF) – National Research Council of Italy (CNR), via Piero Gobetti 101, Bologna 40129, Italy

[c] Institute of Nanostructured Materials (ISMN) – National Research Council of Italy (CNR), via Piero Gobetti 101, Bologna 40129, Italy

[d] Centro Ceramico, via Valle D'Aosta 1, Sassuolo, MO 41049, Italy

[e] Department of Mechanical Engineering, Chalmers University of Technology, Hörsalsvägen 7B, Gothenburg 412 96, Sweden

* Corresponding author: giacomo.foli2@unibo.it

Gas Transport analysis

All gas transport analyses were performed at 35 °C according to a manometric technique reported in ASTM D 1434. All tested samples had an area of 2.2 cm^2 and applied Δp was in the range of 1.7 - 1.9 bar; higher Δp, c.a. 4 bar, were only used in the case of methane in order to increase the flux.

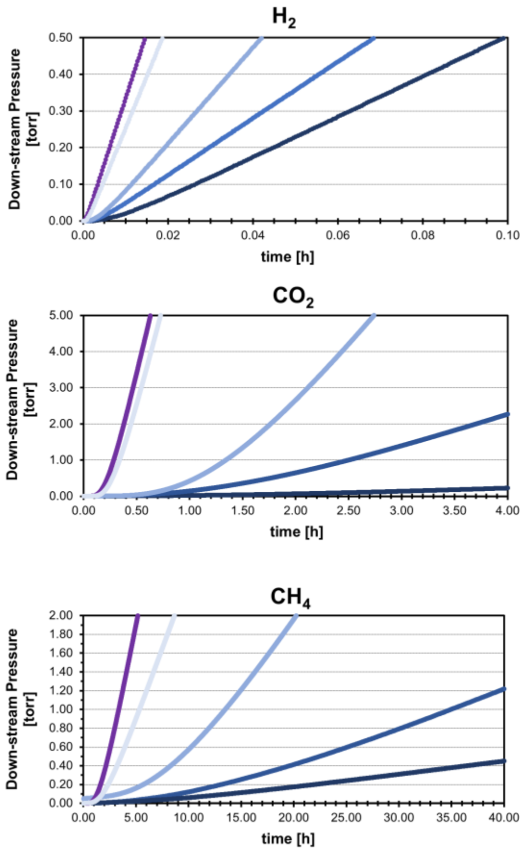

Figure S1. Output of typical permeation experiments performed with the three penetrants through all the fabricated membranes: purple lines are the transient curves of the polyimide substrate while blue lines (from lighter to darker) are the transient curves for the polyimide substrate coated with 1, 3, 5 and 10 cycles of deposition membranes; the steady-state slopes are used for the calculation of the permeance.

Calculation of the permeances of the membranes

All the prepared membranes were build-up on a polyimide substrate. Such prepared composites, i.e. substrates coated with different membranes, were tested as above described, and the overall permeances were determination. The permeances of the membranes only were calculated by applying the equation of the resistances in series and by knowing the permeation properties of polyimide substrate only. Indeed, the permeances of gases are the reciprocal of the resistances to transport of corresponding gases. Thus, in a composite composed of a polyimide substrate coated with a general membrane, permeances of gases of the membrane only can be calculated from **Equation S1**:

$$\frac{1}{Q(g)_{tot}} = \frac{1}{Q(g)_{PI}} + \frac{1}{Q(g)_m} \qquad \textbf{Equation S1}$$

where g is a generic penetrant, $Q(g)_{tot}$ is the permeance of the generic penetrant g trough the polyimide/membrane composite and $Q(g)_{PI}$ is the permeance of the generic penetrant g trough the neat polyimide.

The permeance of the generic penetrant g trough the membrane only, $Q(g)_m$, could be calculated according to **Equation S2**:

$$Q(g)_m = \left(\frac{1}{Q(g)_{tot}} - \frac{1}{Q(g)_{PI}}\right)^{-1} \qquad \textbf{Equation S2}$$

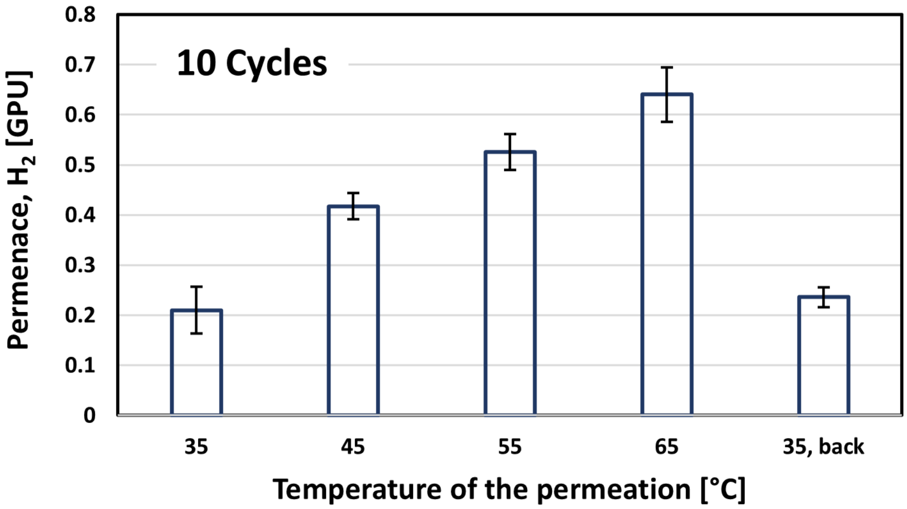

Figure S2. Values of hydrogen permeances through the 10 cycles of deposition membrane at growing temperature.

Table S1. Permeances of the four different membranes, with respect to the three target penetrants: H_2, CO_2 and CH_4. Average values and standard deviations are reported.

**Cycles of deposition**	**#**	**Permeances [GPU]**		
		H_2	CO_2	CH_4
1 cycle	01	19.9	1.16	$2.47 \cdot 10^{-2}$
	02	22.4	1.73	$3.07 \cdot 10^{-2}$
	03	22.1	1.68	$3.00 \cdot 10^{-2}$
	**Av.**	(21.5 ± 1.4)	(1.53 ± 0.32)	$(2.85 \pm 0.33) \cdot 10^{-2}$
3 cycles	01	2.76	0.480	$4.96 \cdot 10^{-3}$
	02	3.06	0.543	$6.01 \cdot 10^{-3}$
	03	2.98	0.501	$5.12 \cdot 10^{-3}$
	**Av.**	(2.93 ± 0.16)	(0.508 ± 0.032)	$(5.36 \pm 0.57) \cdot 10^{-3}$
5 cycles	01	0.486	$2.03 \cdot 10^{-2}$	$4.44 \cdot 10^{-4}$
	02	0.449	$1.56 \cdot 10^{-2}$	$4.12 \cdot 10^{-4}$
	03	0.438	$1.51 \cdot 10^{-2}$	$4.08 \cdot 10^{-4}$
	**Av.**	(0.458 ± 0.025)	$(1.70 \pm 0.28) \cdot 10^{-2}$	$(4.21 \pm 0.20) \cdot 10^{-4}$
10 cycles	01	0.160	$5.37 \cdot 10^{-3}$	$2.02 \cdot 10^{-4}$
	02	0.218	$9.16 \cdot 10^{-3}$	$2.54 \cdot 10^{-4}$
	03	0.253	$6.11 \cdot 10^{-3}$	$2.10 \cdot 10^{-4}$
	**Av.**	(0.210 ± 0.047)	$(6.88 \pm 2.01) \cdot 10^{-3}$	$(2.22 \pm 0.28) \cdot 10^{-4}$

Imaging of the top surfaces of all the fabricated membranes

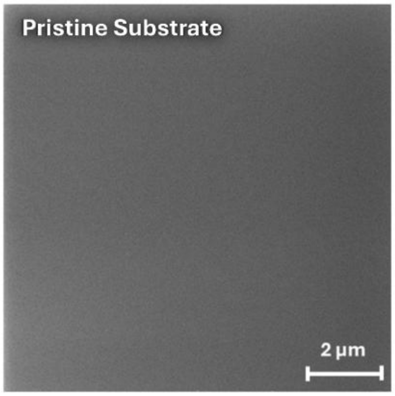

Figure S3. SEM imaging of the pristine substrate (flat polyimide substrate) used for the growth of our membranes.

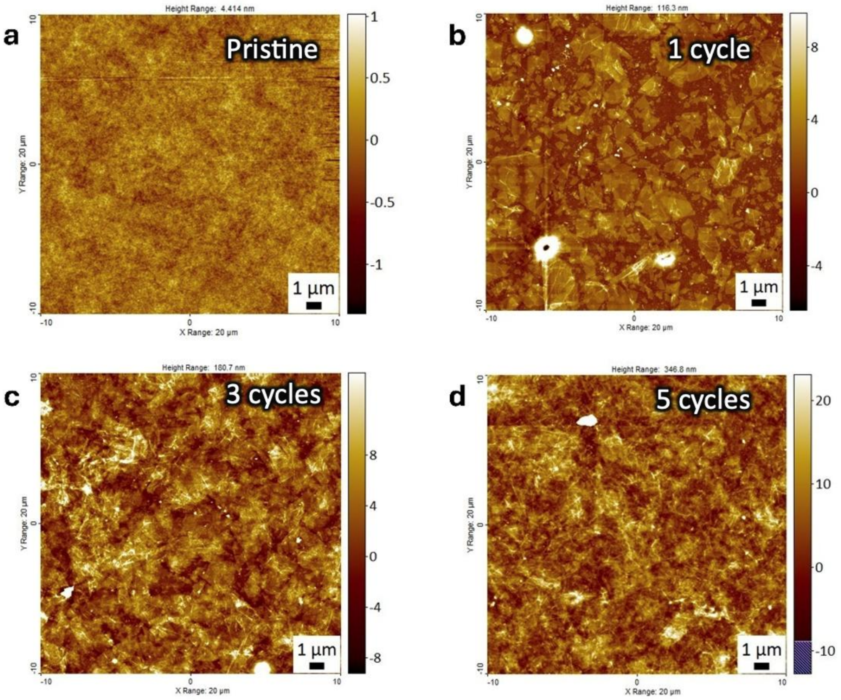

Figure S4. AFM imaging of a) the pristine substrate (flat polyimide substrate) used for the growth of our membranes and top surface of the membranes after b) 1, c) 3 and d) 5 cycles of deposition.

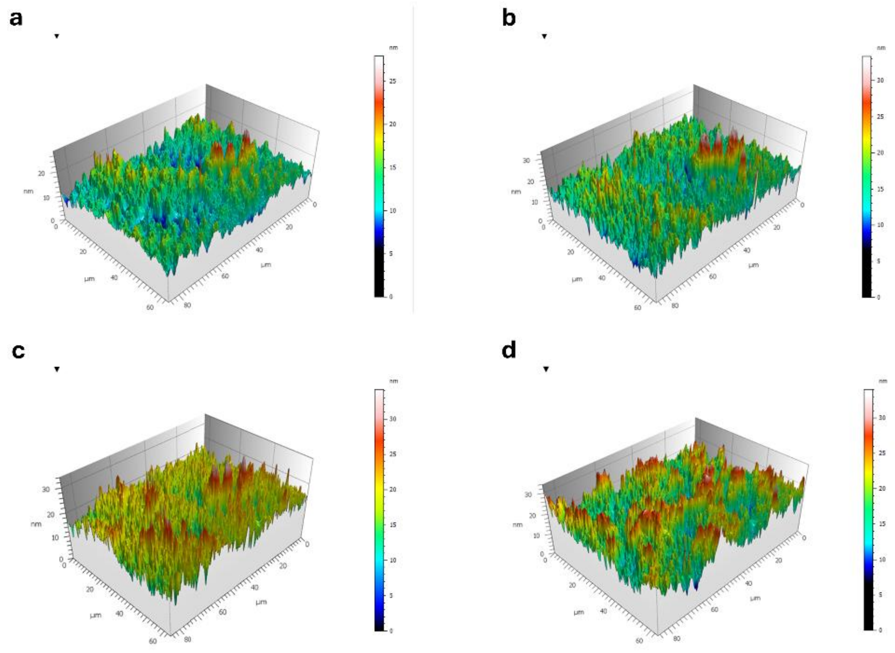

Figure S5. Optical Profilometry imaging of a) the pristine substrate (flat polyimide substrate) used for the growth of our membranes and top surface of the membranes after b) 1, c) 3 and d) 5 cycles of deposition.

QCM analyses

The shift in frequency of the quartz crystal was determined at controlled temperature (25 °C) and relative humidity (30-40% R.H.). The conversion of all recorded shifts in frequency to corresponding changes in mass were achieved using the Sauerbrey equation.[1] In our specific case, the applied formula was:

$$\Delta f = -649.8 \cdot \Delta m \qquad \textbf{Equation S3}$$

where Δf is the shift in frequency recorded by the instrument, expressed in $[Hz]$, and Δm is the calculated variation in mass, expressed in $[\mu g]$. All the corresponding grammages, specific mass $[\mu g \cdot cm^{-2}]$, were calculated dividing mass $[\mu g]$ over the active area of the quartz crystal $[cm^2]$.

Table S2. Stepwise increase in grammage recorded for three independent 10 cycles of deposition membranes, growth on QCM spin coated crystals. Average values and standard deviations are reported.

Cycle of Self-Assembly	Deposited species	Stepwise increase in Grammage [$\mu g \cdot cm^{-2}$]			
		01	02	03	Av.
1	PDDA	0.164	0.106	0.066	(0.112 ± 0.049)
	graphene oxide	0.467	0.763	0.750	(0.660 ± 0.167)
2	PDDA	0.191	0.132	0.176	(0.167 ± 0.031)
	graphene oxide	0.242	0.617	0.573	(0.478 ± 0.205)
3	PDDA	0.577	0.088	0.132	(0.110 ± 0.031)
	graphene oxide	0.622	0.895	0.520	(0.679 ± 0.193)
4	PDDA	0.154	0.203	0.097	(0.151 ± 0.053)
	graphene oxide	0.659	1.107	0.485	(0.750 ± 0.321)
5	PDDA	0.269	0.176	0.163	(0.202 ± 0.058)
	graphene oxide	0.507	1.191	0.899	(0.866 ± 0.343)
6	PDDA	0.163	0.212	0.317	(0.230 ± 0.079)
	graphene oxide	0.842	0.494	0.615	(0.650 ± 0.177)
7	PDDA	0.146	0.128	0.110	(0.127 ± 0.018)
	graphene oxide	0.247	0.653	0.494	(0.464 ± 0.204)
8	PDDA	0.066	0.101	0.119	(0.096 ± 0.027)
	graphene oxide	0.860	0.622	0.494	(0.658 ± 0.186)
9	PDDA	0.154	0.141	0.123	(0.140 ± 0.015)
	graphene oxide	0.639	0.679	0.397	(0.571 ± 0.153)
10	PDDA	0.053	0.075	0.154	(0.094 ± 0.053)
	graphene oxide	0.348	0.551	0.869	(0.589 ± 0.262)

Thicknesses of the membranes determined by AFM

The thicknesses of the membranes were determined using Atomic Force Microscopy (AFM). Each sample was marked with a lateral line made by a homemade Sn engraver (softer than Si) to measure the film thickness relative to the Si substrate. For each sample, 10 images were recorded and analyzed using SPIP software, with a histogram generated for each image (**Figure S6**). An average thickness value was calculated per image, and a final thickness value was obtained by averaging the values from all 10 images. Subtraction of the thickness of the polyimide substrate (**Figure S7a**) allows calculation of the thicknesses of the various membranes, reported in **Table S3**.

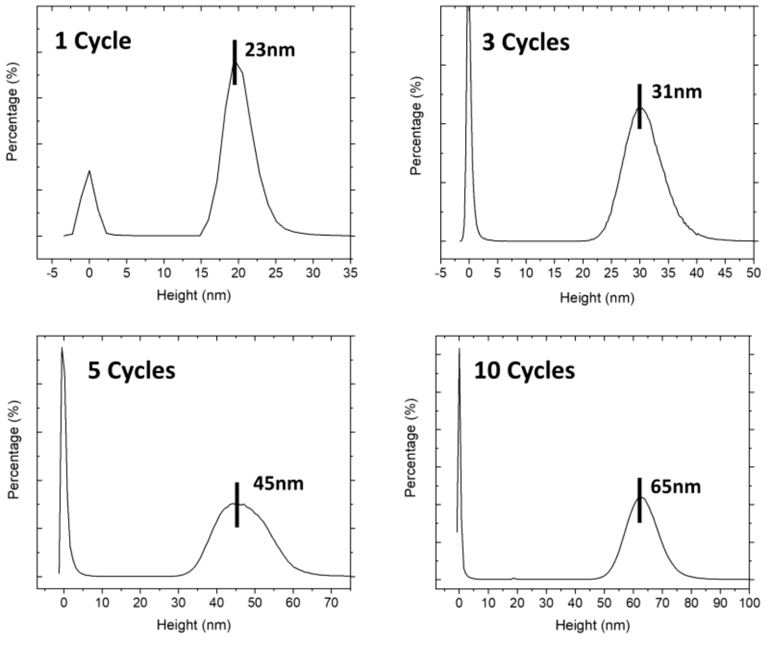

Figure S6. Representative examples of histograms used for the calculation of the thickness of the membrane prepared after 1, 3, 5 and 10 cycles of deposition.

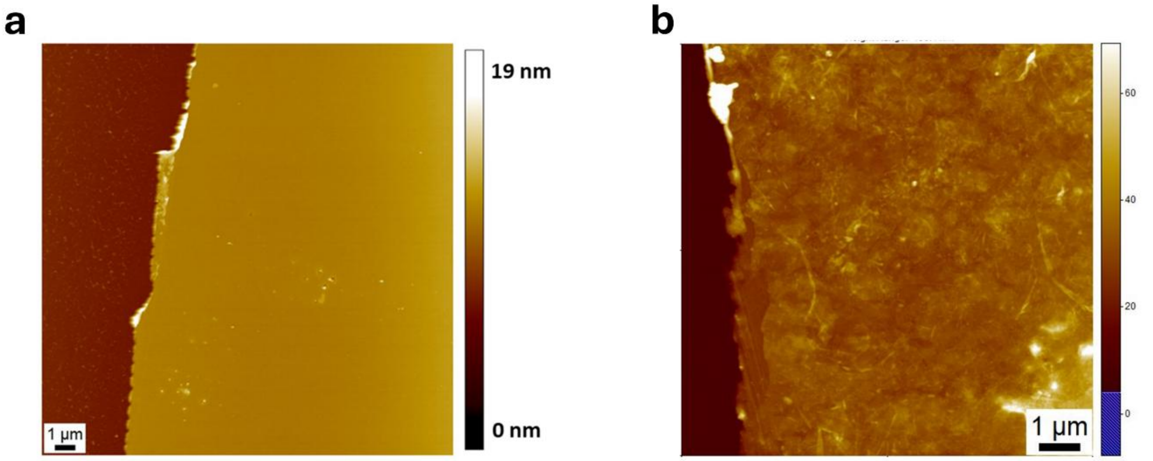

Figure S7. AFM images of a) the scratched flat polyimide substrate and b) the scratched 9.5-cycles architecture deposited on the flat polyimide substrate.

Table S3. Effective thickness measure in all the scratched samples and values rescaled according to the number of cycles of deposition; the error on the Total Thickness correspond to the surface roughness calculated from AFM data (RMS, Table 1).

	**Substrate**	**1 cycle**	**3 cycles**	**5 cycles**	**9.5 cycles**	**10 cycles**
**Total Thickness, [nm]**	19.8 ± 0.8	23.0 ± 1.4	30.9 ± 2.7	38.5 ± 4.3	54.53 ± 5.4	66.50 ± 5.5
**Membrane Thickness, [nm]**	-	3.2 ± 2.2	11.1 ± 3.5	18.7 ± 5.1	34.7 ± 6.2	46.7 ± 6.3
**Normalized Thickness, [nm/cycle]**	-	3.20 ± 2.21	3.70 ± 1.17	3.74 ± 1.01	3.66 ± 0.65	4.67 ± 0.58

ζ-potential of the surface of the membranes

All ζ-potentials were recorded at pH = 7 in a $KCl_{(aq)}$, 1mM with a SurPASS 3 (Anton Paar) equipped with an Adjustable Gap Cell (dimension: 10x20 mm).

Table S4. Surface ζ-potential recorded for the membranes. Every value was measured on an independently self-assembled membrane. Average values and standard deviations are reported.

Cycles of Self-Assembly	ζ-potential (this work) [mV]	ζ-potential (previous work)[2] [mV]
1	-69.3	-45.1
3	-53.6	-39.4
5	-58.7	-32.3
10	-51.4	-34.2
Av.	-58.3 ± 8.0	-37.8 ± 5.7

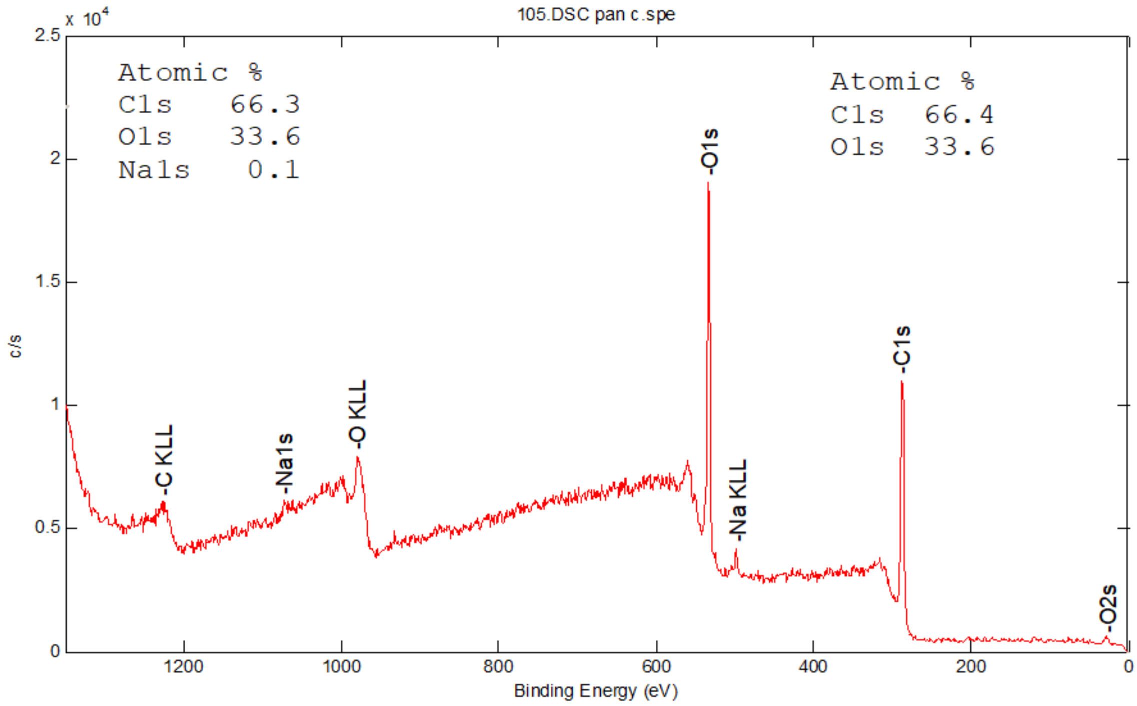

Figure S8. XPS spectrum of the GO used in this work (LayerOne) and the atomic O/C ratio.

XRD analyses

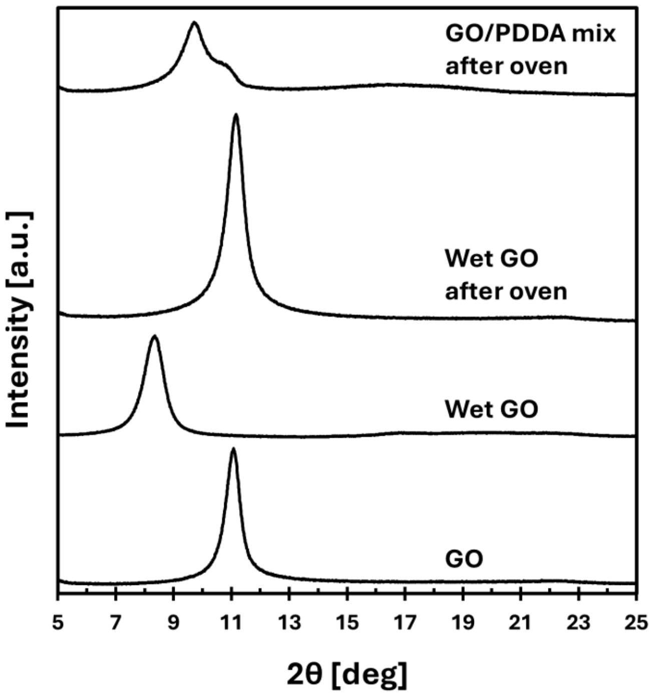

Figure S9. Comparison among XRD patterns of, from bottom to top, pristine GO used in this work (LayerOne), GO swollen in water before and after oven drying and GO dispersed in a $PDDA_{(aq)}$ 10 wt.% solution, separated by centrifugation and dried in vacuum-oven at 35 °C for 10 days.

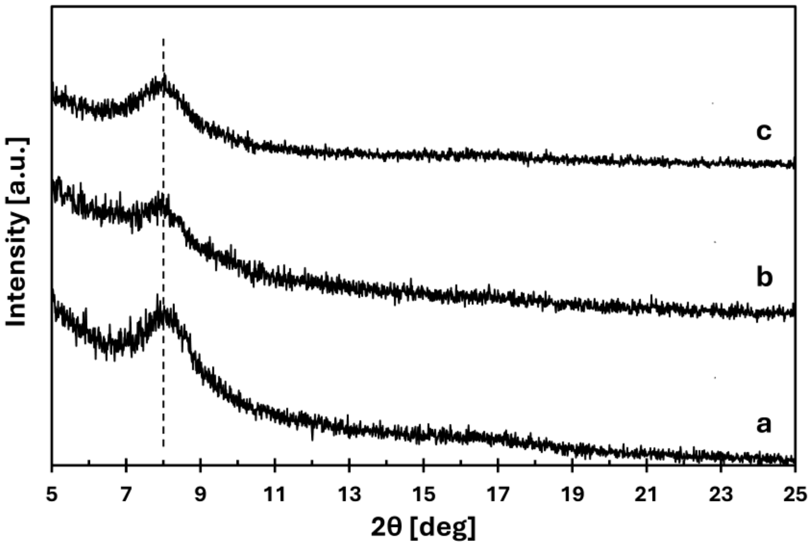

Figure S10. Comparison among XRD patterns of our membrane a) after 30 minutes of IR irradiation, b) after successive 10 days of vacuum at 35 °C and c) after successive 1 hour of vacuum at 100 °C.

Table S5. d-spacing extracted from the XRD patterns for all the prepared membranes and the coherence length, L', calculated using the Scherrer's formula. Peak area is also reported.

**Cycles of self-assembly**	**2θ [deg]**	**d-spacing [nm]**	**L' [nm]**	**Peak Area [cps]**
1 cycle	-	-	-	-
3 cycles	8.10 ± 0.01	1.09 ± 0.08	4.30 ± 0.03	1700 ± 100
5 cycles	8.10 ± 0.01	1.09 ± 0.06	4.70 ± 0.03	2900 ± 100
10 cycles	8.07 ± 0.01	1.09 ± 0.06	5.00 ± 0.03	5000 ± 100

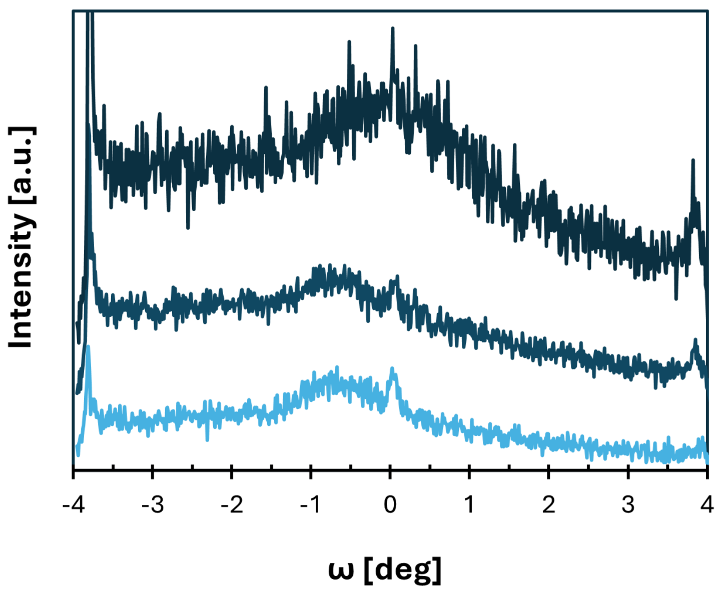

Figure S11. Rocking curves recorded at 2θ = 8° for the fabricated membranes, from bottom to up: 3, 5 and 10 cycles of deposition. Rocking curve for the 1 cycle of deposition membrane was not recorded due to absence of XRD pattern.

Reference

(1) Baba, A.; Kaneko, F.; Advincula, R. C. Polyelectrolyte Adsorption Processes Characterized in Situ Using the Quartz Crystal Microbalance Technique: Alternate Adsorption Properties in Ultrathin Polymer Films. *Colloids Surf. A Physicochem. Eng. Asp.* **2000**, *173* (1-3), 39-49. DOI: 10.1016/S0927-7757(00)00579-3

(2) Foli, G.; Kovtun, A.; Liscio, F.; Battaglini, E.; Ligi, S.; Fiorini, M.; Minelli, M.; Palermo, V. Tuneable Permeability to H2, CO2, He, and Ar in Graphene Oxide−PDDA Self-Assembled Multilayers, Yielding Good Selectivity at High Flux. *Adv. Mater. Interfaces* **2024**, *11* (2), 2300357. DOI: 10.1002/admi.202300357
